# Single Cell Detection of the p53 Protein by Mass Cytometry

**DOI:** 10.3390/cancers12123699

**Published:** 2020-12-09

**Authors:** Oda Helen Eck Fagerholt, Monica Hellesøy, Stein-Erik Gullaksen, Bjørn Tore Gjertsen

**Affiliations:** 1 Centre for Cancer Biomarkers (CCBIO), Department of Clinical Science, University of Bergen, 5007 Bergen, Norway; ofa043@uib.no; 2 Department of Internal Medicine, Hematology Section, Haukeland University Hospital, 5021 Bergen, Norway; monica.hellesoy@helse-bergen.no (M.H.); stein.gullaksen@uib.no (S.-E.G.)

**Keywords:** p53, mass cytometry, antibodies

## Abstract

**Simple Summary:**

Investigation of protein expression in cancer cells is an important part of the diagnostic process. Increasing knowledge about expression of different proteins has been exploited for prognostic assessments and in some cases also for selection of treatment. The p53 protein has proven important in development of various cancers, and the expression of this protein and its signaling pathway is therefore of interest when examining cancer patient samples. Here, we present mass cytometry as a tool for detection of p53 expression. Mass cytometry allows for measurement of up to 50 parameters per sample with single cell resolution, and we aim to demonstrate its potential for p53-focused research.

**Abstract:**

Purpose: The p53 protein and its post-translational modifications are distinctly expressed in various normal cell types and malignant cells and are usually detected by immunohistochemistry and flow cytometry in contemporary diagnostics. Here, we describe an approach for simultaneous multiparameter detection of p53, its post-translational modifications and p53 pathway-related signaling proteins in single cells using mass cytometry. Method: We conjugated p53-specific antibodies to metal tags for detection by mass cytometry, allowing the detection of proteins and their post-translational modifications in single cells. We provide an overview of the antibody validation process using relevant biological controls, including cell lines treated in vitro with a stimulus (irradiation) known to induce changes in the expression level of p53. Finally, we present the potential of the method through investigation of primary samples from leukemia patients with distinct *TP53* mutational status. Results: The p53 protein can be detected in cell lines and in primary samples by mass cytometry. By combining antibodies for p53-related signaling proteins with a surface marker panel, we show that mass cytometry can be used to decipher the single cell p53 signaling pathway in heterogeneous patient samples. Conclusion: Single cell profiling by mass cytometry allows the investigation of the p53 functionality through examination of relevant downstream signaling proteins in normal and malignant cells. Our work illustrates a novel approach for single cell profiling of p53.

## 1. Introduction

The transcription factor and tumor suppressor p53 is commonly referred to as “the guardian of the genome” and is an essential player in the regulation of genotoxic and stress-induced apoptosis. In healthy cells, the level of p53 is kept low by the negative regulator murine double minute 2 protein (Mdm2) [1]. As a response to cellular stress or other conditions threatening genomic stability, the level of p53 rises and the p53 signaling pathway is subsequently activated. p53 exerts its function by acting as a transcription factor, and occasionally a transcriptional repressor, for specific target genes [2]. The resulting outcome of pathway activation includes cell cycle arrest in G1/S and G2/M to orchestrate repair of DNA damage, or, if repair is not possible, induction of apoptosis [3]. Mdm2 and p53 interact in a reciprocal negative feedback-loop; where p53 induces Mdm2 transcription, and Mdm2 in turn blocks the p53 transactivation domain and targets p53 for ubiquitin-dependent degradation [4]. In a setting where *TP53* is mutated, the expression level of the (mutant) p53 protein is elevated. This is largely due to the absence of activation of a p53 downstream response, including transactivation of Mdm2, which leads to stabilization and accumulation of the mutated p53 protein [5].

The frequency of *TP53* mutations in malignancies span from approximately 90% in ovarian cancers to <10% in acute myeloid leukemia (AML) [6]. Even though only a minority of AML patients have a *TP53* mutation, those harboring a mutation usually exhibit resistance to chemotherapy and have a poor prognosis [7]. Moreover, the expression of various p53 isoforms is modulated by chemotherapy and has been shown to correlate with therapy response in this malignancy [8].

p53 is subject to an extensive range of post-translational modifications that are important for the stability of the protein [9]. The N-terminal of p53 is particularly subject to phosphorylation, while the C-terminal is subject to modulations such as acetylation, methylation, and ubiquitylation [1]. An important effect of such modifications is preventing p53 from achieving optimal interaction with Mdm2, which under unperturbed circumstances is responsible for the degradation of p53 [10].

Immunohistochemistry and western blot are considered the gold standard for detection of p53.

However, western blot only reveals bulk cell characteristics and requires relatively large amount of material. Immunohistochemistry is employed in certain malignancies as part of the diagnostic work up [11,12], and can be used to determine the relative abundance of p53 protein in single cells. For instance, in malignant pre-leukemic myelodysplasia, immunohistochemistry has revealed protein expression of p53 and its related proteins in hematopoietic progenitor cells. This may be related to the pathogenesis of this heterogeneous bone marrow malignancy, and has been shown to provide important prognostic information [13,14,15]. Flow cytometry offers simpler and faster sample processing, and also the possibility of analyzing cellular subsets based on the expression of surface markers. However, traditional flow cytometry is limited in the number of parameters measured on each single cell (typically between 5–15) due to fluorescence spillover and endogenous cell auto-fluorescence.

Mass cytometry is a relatively novel platform capable of measuring up to 50 parameters on each single cell, by combining metal isotope-bound antibodies with mass spectrometry detection [16,17]. By replacing the fluorophores used in flow cytometry with heavy metal isotopes, the obstacle of signal overlap is circumvented. As the metals conjugated to antibodies are not present in biological systems, background signals are negligible, and the problem of endogenous autofluorescence is similarly eliminated. This makes mass cytometry an ideal tool for simultaneous intracellular pathway analysis and functional assessments in single cells. Furthermore, the use of cell surface markers allows for the investigation of such processes in distinct cell types. In addition, the technology allows for barcoding and multiplexing of unique samples, allowing direct comparison of quantitative data collected from highly heterogeneous samples, and the possibility of including internal reference samples in every run [16,18,19].

Although p53 in mass cytometry was briefly explored previously [20], here we present mass cytometry as a tool that enables assessment of the p53 expression, including post-translational modification (acetylation at K382) and downstream targets, in various cancer cells, focusing on the aggressive bone marrow-derived cancer AML.

## 2. Results

### 2.1. Titration and Validation of p53-Specific Antibodies for Mass Cytometry

To explore p53 expression levels in single cells by mass cytometry, we used three p53-specific antibodies; p53 (clone: DO-7), mutant p53 (clone: Y5, hereafter referred to as p53^mut^), and K382 acetylated p53 (clone: REA529, hereafter referred to as ac-p53 [K382]). These antibodies were selected based on previous experiences from flow cytometry and the vendor’s possibility to deliver the antibodies in suitable carriers compatible for conjugation to metal isotopes. The epitope of the DO-7 antibody is amino acid (aa) residues 37–45 of the N-terminal. The Y5 antibody is mutant-specific, with an epitope at aa 1–100. The REA529 antibody detects p53 acetylated in the C-terminal region at K382.

In the conjugation process, partial reduction of antibodies is required [21]. Thus, validation of the performance of the antibodies post-conjugation is necessary. Furthermore, optimal antibody concentration depends on multiple factors (e.g., sample type, antibody lot and staining protocol), and must therefore be experimentally determined by titration. Validation and titration were performed in parallel by six serial antibody dilutions, as previously described [22]. Importantly, excessive antibody concentration might yield unspecific antibody binding and false positive signals. Thus, including both positive and negative biological controls in all antibody titration experiments is essential. Since the mass cytometry technology allows for barcoding and multiplexing samples, it is possible to include both positive and negative controls combined in the same staining.

Here, we included five cell lines as biological controls; HL60 (*TP53*-/-), MOLM13 (*TP53* wt), NB4 (*TP53* mut), OV90 (*TP53* mut) and Raji (*TP53* mut) (Table 1). In addition, the validation experiment also included MOLM13 exposed to γ-irradiation (25 Gy), which is known to cause stress-induced p53 up-regulation [23,24]. All control cells used for titration and validation were combined in a single barcoded pooled sample, allowing direct comparison of antibody signal across all samples simultaneously for each titer.

**Figure 1:**
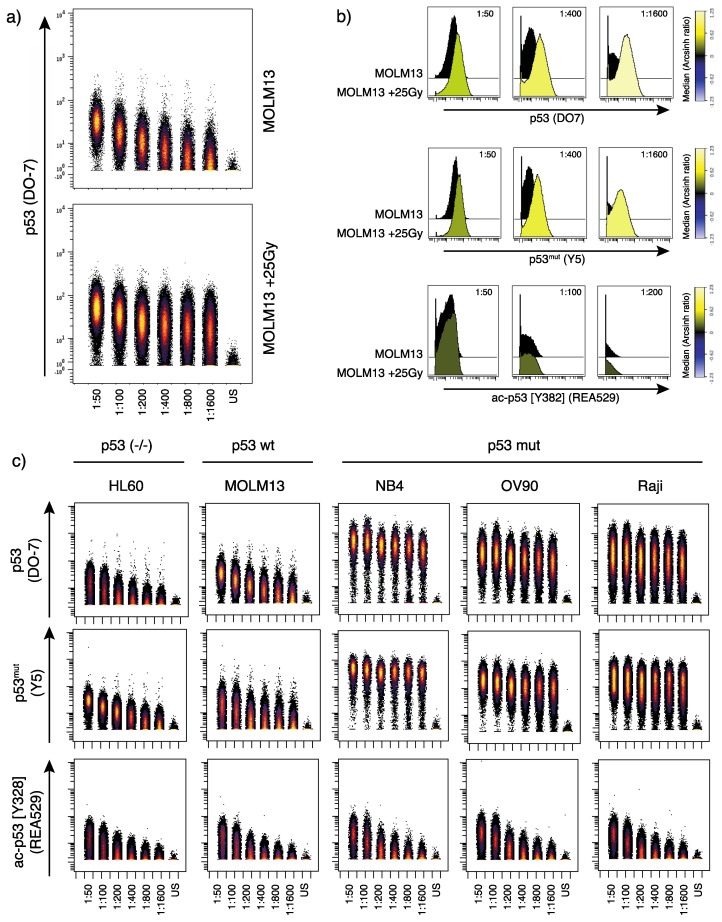
Serial dilution of p53-specific antibodies. (**a**) Dot plot showing p53 (DO-7) signal in MOLM13 cells (untreated; top and γ-irradiated (25Gy); bottom) detected by decreasing antibody concentrations (left to right, and unstained (US) cells at the far right). (**b**) Histograms showing p53 induction (detected by the DO-7 and Y5 antibodies; upper and middle panels) and p53 acetylation (lower panel) in MOLM13 cells (+/− γ-irradiation, 25Gy) at the indicated dilutions. Color scale indicates signal intensity (median dual counts) presented as arcsinh ratio (relative to control). (**c**) Dot plot showing titration of p53 (DO-7), p53^mut^ (Y5) and ac-p53 [K382] (REA529) antibodies in HL60, MOLM13, NB4, OV90 and Raji cell lines. *TP53* mutation status of the respective cell lines is indicated above.

Our results showed that the in-house conjugated p53 (DO-7) antibody detected wild type p53 expression in MOLM13 cells, in addition to changes of the p53 expression level induced by γ-irradiation (Figure 1a,b). As we can directly compare antibody staining between samples, we observe that the highest p53 (DO-7) antibody titers yield signal in the HL60 cells (Figure 1c), indicating unspecific binding. Therefore, a low antibody concertation (1:400) was found to be optimal in this experiment. In the process of deciding an optimal antibody concentration, assessment of channels prone to signal spillover is of importance. In mass cytometry, spillover is predictable and can thus be assessed and partly accounted for by designing appropriate panels and selecting appropriate antibody concentrations for downstream experiments [22]. Furthermore, the p53 (DO-7) antibody also detected p53 expression in the *TP53* mutant cell lines NB4, OV90 and Raji, which is in concordance with previous reports [29].

The p53^mut^ (Y5) antibody showed as expected a strong signal in the *TP53* mutated cell lines NB4, OV90 and Raji (Figure 1c). However, this antibody also unexpectedly produced a signal in the *TP53* wild type MOLM13 cells and *TP53* negative HL60 cells, which could suggest unspecific antibody binding. Furthermore, we observe an induction of wild type p53 following γ-irradiation using this antibody, indicating that the antibody indeed also recognizes wild type p53 (Figure 1b).

The ac-p53 [K382] (REA529) antibody in general showed low signal with comparable staining patterns across all samples tested (Figure 1c). However, we found a very modest signal increase in MOLM13 cells after γ-irradiation (Figure 1b), interpreted as specific detection of irradiation-induced acetylation at this residue. Increased acetylation of p53 at acK382 in response to irradiation has been shown previously [30].

**Figure 2:**
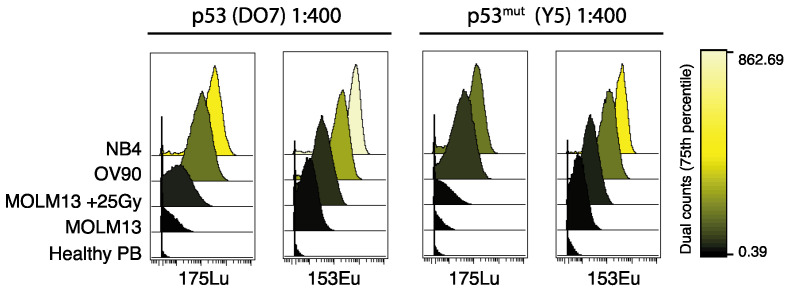
Signal generated by two p53 antibodies conjugated to the 153Eu and 175Lu isotopes, at 1:400 dilution. Histograms of signal from p53 (DO-7) (left panels) and p53^mut^ (Y5) antibodies (right panels) from NB4, OV90 and MOLM13 (+/− γ-irradiation, 25Gy) cell lines, and in healthy peripheral blood. Color scale indicates signal intensity (dual counts, 75th percentile).

It is known that different metal tags for mass cytometry generate different signal intensities due to tuning of instrumental ion-optics. Importantly, the variation in sensitivity across the mass range is instrument-specific [31]. Thus, selection of channels can be decisive for the data quality, particularly for signals generated by low abundance targets. We illustrate this by comparing the signals generated by two antibodies; p53 (DO-7) and p53^mut^ (Y5), each conjugated to two different isotopes. 50 μg of the respective antibodies was conjugated to both the 153Eu and the 175Lu isotope. The conjugation was performed in parallel and with equal initial antibody concentration. The following antibody staining and analysis was performed simultaneously to minimize potential causes of difference in signal intensity. Overall, we found that the 153Eu conjugate of both antibodies generated a stronger signal in the same sample, as compared to the same initial antibody concentration of the respective 175Lu conjugate, in our mass cytometer (Figure 2). Thus, our data support previous recommendations that antibodies detecting low abundance targets (such as p53 in healthy unperturbed cells) should be conjugated to the high-sensitivity isotopes if possible, while the remaining isotopes should be used for antibodies detecting more abundant targets, like surface markers [22]. We also recommend validation and titration of the antibodies specifically on the mass cytometer on which the antibodies will be further used.

### 2.2. The Potential of Mass Cytometry for Detection of p53 and p53-Related Proteins in Primary Samples

To illustrate the potential of mass cytometry, we analyzed primary samples from patients with hematological malignancies using a panel of antibodies designed to detect different immunophenotypes and intracellular proteins in the p53 network. Our patient cohort consisted of three AML patients with *TP53* mutation (AML017, AML018 and AML306), one AML patient with *TP53* wild type (AML023), and two patients with B-cell malignancies; one hairy cell leukemia (HCL089) and one chronic lymphocytic leukemia (CLL151). In addition, we included a reference sample made by pooling peripheral blood from healthy donors (HDPB). *TP53* status of the primary samples is summarized in Table S1.

**Figure 3:**
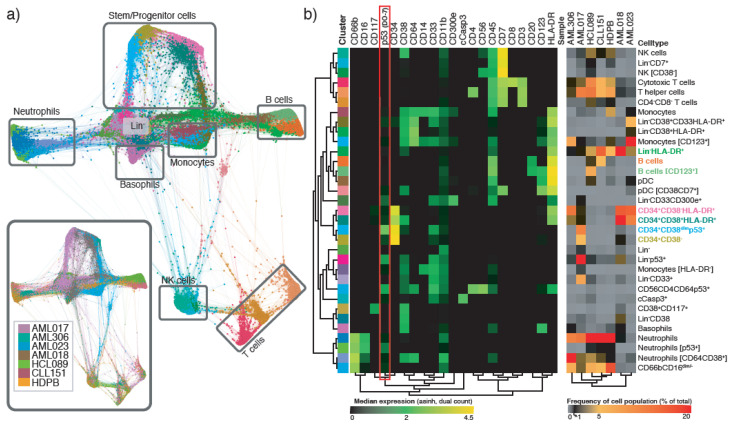
Clustering analysis of primary leukemic samples revealing distinct immunophenotypes. (**a**) Force directed layout showing all clusters detected by x-shift in all samples, both cell type (33 cell types) and sample (inset; AML017, AML018, AML306, AML023, HCL089, CLL151 and HDPB) is color-coded. The immunophenotype of cell subsets was determined by manual evaluation and major cell types are indicated in gray boxes. (**b**) The clusters are color-coded corresponding to cell type in a. Heatmap showing the phenotype of all 33 cell clusters (left, median marker expression, arcsinh of dual count), and the frequency of the different cell clusters in the seven individual primary samples (right, percent of all cells in the respective sample).

The samples were barcoded and pooled in a single sample, and subsequently stained with an antibody panel consisting of 19 surface markers and 16 intracellular markers (Table S2). Single cell data from all samples were pooled and clustered using the x-shift algorithm [32], where we included all the surface markers in addition to p53 (DO-7) for clustering. p53 was included to enable investigation of specific populations defined by p53 expression. Manual inspection, annotation and merging of clusters resulted in the identification of 33 immunophenotypically distinct cell types, including three populations characterized by high p53 expression (Figure 3a,b, Table S3). Two of these populations were mainly confined to one single patient; AML017. This included a Lin^−^p53^+^ population (31% of total cell count), and a CD34^+^CD38^dim^p53^+^ population (12% of total cell count). Approximately 3% of the cells in patient sample AML018 was also fund in the CD34^+^CD38^dim^p53^+^ population. In our small cohort including three *TP53* mutated AML patients, we found that the patient with the highest variant allele frequency (VF) also exhibited the highest p53 (DO-7) expression. In general, we observed that p53 was mostly expressed in CD34^+^ or Lin^−^ populations (Figure 3b). However, we also identified a third p53+ population, which was a small population of neutrophils where p53 (dim) and CD11b expression separated it from the main neutrophil population. This small population was observed in the HCL089 and CLL151 patient samples, and also in the HDPB sample.

**Figure 4:**
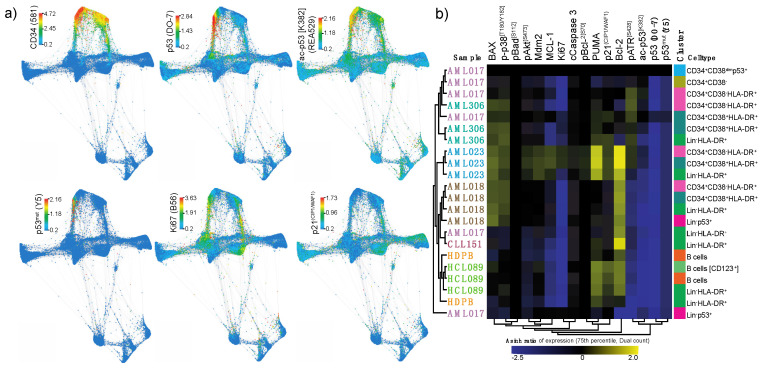
p53 network in primary samples. (**a**) Force directed layout showing the single cell expression level (arcsinh transformed dual counts) of CD34 (581), p53 (DO-7), ac-p53 [K382] (REA529), p53^mut^ (Y5), Ki67 (B56) and p21^(CIP1/WAF1)^ (12D1). (**b**) Heatmap showing the relative expression level of intracellular signaling proteins in selected cell populations. Clusters are color-coded corresponding to the color of the cluster name in Figure 3a. Color scale indicates expression level (arcsinh ratio) relative to the CD34^+^CD38^dim^p53^+^ population in patient AML017 (top row).

Force directed layout visualization of single cell data revealed an association between high p53 (DO-7) expression and ac-p53 [K382] (REA529), p53^mut^ (Y5) and Ki67 (Figure 4a). To further evaluate differences in p53 network activation, we compared the expression levels of all intracellular markers in selected populations in different samples to the CD34^+^CD38^dim^p53^+^ population from AML017 (Figure 4b). By performing a relative analysis (arcsinh ratio) using this population as reference, patterns in p53 network activation distinguished by p53 expression could be revealed. Most strikingly, we found that the expression of Ki67, a maker of proliferation, appeared to correlate well with the p53 expression level. We also observed that cell populations from AML023, the only *TP53* wild type patient, showed a highly distinct p53 network pattern. This is likely due to the high expression levels of PUMA and p21^(CIP1/WAF1)^, relative to the CD34^+^CD38^dim^p53^+^ population in AML017. We interpret this as a consequence of functional p53 protein in this patient, where low levels of p53 is able to activate downstream targets.

**Figure 5:**
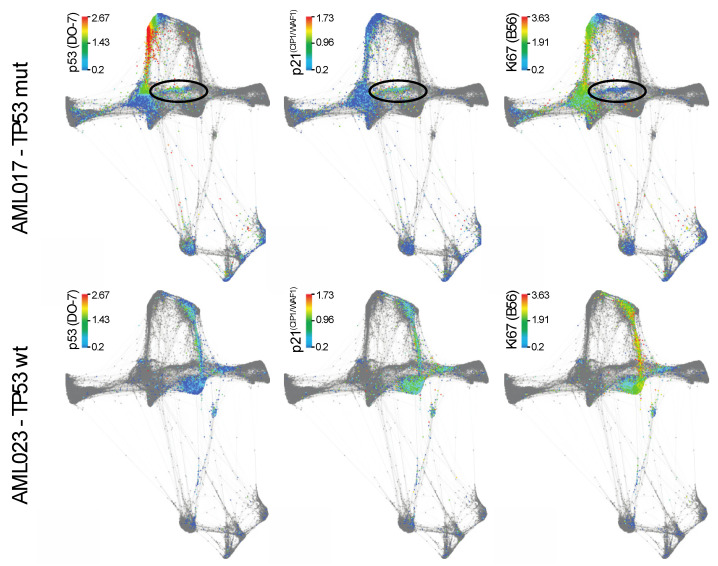
Force directed layout analysis of AML017 (upper panel) and AML 023 (bottom panel). Single cell data from patient AML017 and AML 023 showing the expression of p53 (DO-7), p21^(CIP1/WAF1)^ (12D1) and Ki67 (B56).

Individual analysis of the different patients using viSNE can be found in Figure S1 [33]. As AML017 showed the particularly interesting feature of expressing the highest level of p53, we decided to focus on this *TP53* mutated patient. We investigated the force directed layout by visualizing the cells originating solely from patient AML017 (Figure 5, upper panel). The *TP53* wild type patient AML023 was also included for comparison (Figure 5, bottom panel). In AML017 the majority of the malignant population expressed high levels of p53, while a distinct minor population (Lin^−^HLA-DR^+^) was characterized by lower p53 expression. By assessing the expression of the p53 downstream target p21^(CIP1/WAF1)^ and the proliferation marker Ki67, we observed a seemingly inverse correlation. Indeed, the Ki67 expression was high, while p21^(CIP1/WAF1)^ expression was low. The reversed was observed in the p53 low population (highlighted in circles, Figure 5). In AML023, expression of p53 (DO-7) correlated with the expression of p21^(CIP1/WAF1)^. Ki67 was also expressed in this population.

## 3. Discussion

In this study, we demonstrate the use of mass cytometry to examine p53 expression in cell lines and patient samples of leukemia. Investigation by mass cytometry demands for single cell suspension. Hematological malignancies are therefore particularly suitable, as the samples naturally are in single cell suspension. Thus, peripheral blood and bone marrow do not require extensive processing by mechanical and/or enzymatical degradation as solid tissues demand for. Peripheral blood and bone marrow samples can be fixed shortly after sampling using commercially available reagents (e.g., lyse/fix, BD Biosciences) or proteomic stabilizer (Smart Tube Inc.). This greatly reduces the risk of altering intracellular signaling profiles prior to fixation. Furthermore, due to the possibility to simultaneously perform broad phenotyping and detect expression of multiple intracellular proteins, mass cytometry allows for assessment of protein expression in specific cellular subsets in primary samples. However, limitations of the technology do exist, and these are thoroughly addressed in Spitzer et al. [18]. In short, even though mass cytometry is regarded as an advancement of flow cytometry, the technology is partly limited by slower acquisition time, increased cell loss during sample preparation, and destruction of cells during analysis that precludes single cell sorting post analysis. Additionally, mass cytometry does not include light scatter parameters for assessment of cellular size and granularity.

In contrast to immunoblot analysis of p53 proteins, single cell detection by mass cytometry does not allow discrimination of molecular weight of the p53 species detected. This is especially a challenge in regard to the detection of p53 isoforms. To our knowledge, no isoform-specific antibodies developed have currently been validated to show compatibility with detection of non-denatured proteins by flow- or mass cytometry. Development of such specific antibodies could allow the assessment of biology and functionality of the p53 isoforms. Future development of antibodies with improved specificity could ease the identification of endogenous p53 isoforms, and as mass cytometry allows for high-dimensional data collection, thorough assessment of the individual isoforms’ properties in a natural system could be achieved. As the method’s span of detection relies solely on appropriate probes (usually antibodies), development of specific monoclonal antibodies directed toward specific *TP53* mutations (specific amino acid sequences in mutated *TP53*) could enable mass cytometry to distinguish different mutations. Alternative ways of detecting specific mutations could be to use RNA probes toward specific mutations; a strategy similar to proximity ligation assays for RNA (PLAYR) or metal in situ hybridization (MISH) [34,35].

Detection of p53 using antibodies is limited by the antibody specificity, sensitivity, and affinity to p53 in the particular sample preparation. The p53 protein is encoded by a gene that may be the origin to more than 13 pre-mRNA splicing isoforms [36]. Furthermore, the p53 protein may be processed by DNA-modulated cleavage into specific truncated versions [37]. Finally, the p53 protein can undergo a multitude of post-translational modifications [9]. Thus, the complexity and heterogeneity of the p53 protein structure and functional modifications make antibody specificity, especially in detection of non-denatured protein, a significant challenge. Single cells processed for flow- and mass cytometry typically preserve the protein conformation, in contrast to the denaturating conditions necessary for immunoblot detection. Antibodies suitable for immunoblots might therefore not be compatible with detection of non-denatured protein by cytometric analysis. When choosing antibodies for conjugation to isotopes, it is therefore advisable to select antibodies that have previously been tested and validated by flow cytometry, such as the p53 (DO-7) antibody [38].

Careful use of technical and biological validation is essential for establishing mass cytometry detection, e.g., by a standardized set of cell lines. Similarly, post-translational modifications may be validated by in vitro stimulated cell lines. Here, we demonstrate how *TP53* mutations affect the p53 expression level by using different cell lines for validation experiments. As these cell lines express p53 in varying degrees, we show the importance of titrating antibodies on relevant samples. HL60 cells do not express full-length p53 due to deletion in both alleles [39], while NB4 cells carry a *TP53* mutation, which results in high protein expression [40]. An antibody concentration titrated correctly for MOLM13 and HL60 cells might therefore not be the ideal concentration for NB4 cells. The possibility to multiplex samples for mass cytometry also allows for more extensive use of controls to determine the accuracy of measurement and validation of antibodies. As mass cytometry is a rather novel tool, proper standardization is currently lacking. Identical biological reference sample(s) multiplexed into to each barcode could serve as internal references for the measured parameters. This would ensure comparability across barcodes and could further provide the opportunity to compare datasets acquired on different machines and in different labs [41,42]. Of note is that the first approach toward quantification of mass cytometry output has also recently been published [43]. In this work, a receptor occupancy assay was adjusted to be compatible with the mass cytometry platform. Future approaches toward quantification of protein expression by mass cytometry could employ a similar bead-based strategy, where antigen-coated beads spiked into the sample could be used to calculate a standard curve for antibody binding to the protein target. Thus, quantitative assessment of p53 protein expression could be possible.

In general, mass cytometry is regarded as a less sensitive platform than flow cytometry and low-abundant targets may therefore theoretically be more difficult to detect by mass cytometry. However, the low background signal combined with almost negligible signal spillover in mass cytometry partly compensates for this [44]. The detection limit for the third-generation mass cytometer (Helios) is approximately 350 antibodies per cell, while as low as 40 molecules per cell can theoretically be detected by flow cytometry, when applying the brightest fluorophores [45]. In flow cytometry, the difference in signal intensity may reach 10–50-fold across different fluorochromes, while in mass cytometry, signal strength vary by only 2–4-fold difference dependent of metal mass. However, there is a significant instrument-to-instrument variation [46].

Resting healthy cells express only low levels of p53, and thus, to properly detect p53 by mass cytometry, we argue that careful selection of isotope conjugate during antibody panel design is of importance. This was also evident from our observations when conjugating the same p53 antibodies to two different metal isotopes. Therefore, it is important that the antibodies in a panel are tested and titrated on that exact instrument. We encourage users of mass cytometry to carefully assess the signal generated by the different metal channels, and if feasible, conjugate aliquots of the same antibody to different isotopes for comparison of signal intensity. In our case, the two 153Eu antibody conjugates consistently generated stronger signals than the two 175Lu conjugates in all cell samples expressing low levels of p53 protein. In the cell lines expressing high levels of p53 protein, the signal differences were visually indistinguishable.

We previously compared two-dimensional gel immunoblot with flow cytometry analysis of intracellular proteins in AML [47]. Immunoblot analysis provides a unique window of p53 protein with its isoforms and modifications [8]. However, due to cell heterogeneity in normal and leukemic blood, it is likely that single cell identification and p53 analysis may provide new information about p53 biology. As small cell populations can be decisive in the fate of a malignancy, total cell lysates may not adequately reflect the state of the disease. By applying single cell analytical tools, in-depth investigations of protein expression in subpopulations can be performed.

An increasing number of reports have shown that hematological malignancies may have a minute cell population carrying *TP53* mutations, and that these mutated subpopulations could indicate higher risk of relapse [48,49]. We hypothesize that future single cell diagnostics could incorporate p53 protein analysis to detect these cases. The routine method of identifying *TP53* mutations in clinical diagnostics is sequencing, while loss of *TP53* can be detected by fluorescent in situ hybridization (FISH). Although *TP53* is mutated, the protein can still be translated. The resulting protein product may deviate from normal conformation and thus not retain normal protein function. Downstream targets of p53 might therefore not be affected by p53 activation. Our investigation of patient AML017 exemplifies such an event, where p53 is expressed at high levels in the majority of the malignant population, although the downstream target p21^(CIP1/WAF1)^ remains low. We hypothesize that this pattern is due to the protein product of mutated *TP53* in this patient, which is unable to induce p21^(CIP1/WAF1)^ transcription as a consequence of loss-of-function. However, we also observed a second small population, corresponding to approximately 1% of the cells in this patient, where lower levels of p53 were detected. Here, the expression pattern of p21^(CIP1/WAF1)^ and Ki67 was reversed; a pattern indicative of normal p53 function. This could be due to expression of wild type p53 in this small subpopulation of malignant cells. An alternative explanation could be p53-independent activation of p21^(CIP1/WAF1)^ [50]. Unfortunately, we are not able to conclude on the mutational status of p53 in this population, due to the limitations of p53 antibody specificity. In the *TP53* wild type patient, we observed the expected expression pattern of p53 and p21^(CIP1/WAF1)^. Yet, despite high p21^(CIP1/WAF1)^ expression, the malignant cell population also exhibited high Ki67 expression, indicative of high mitotic activity. An explanation for this inconsistency could be that this patient harbors other driver mutations causing excessive mitotic activity. Numerous other mutations and aberrant signaling pathways in AML have been described to be associated with non-mutational p53 dysfunction [51]. However, we do emphasize that our patient cohort is small, and that AML is a particularly heterogenous malignancy, hampering any decisive conclusion regarding p53 biology.

In our experiments, the p53^mut^ (Y5) antibody unexpectedly yielded signal in both the *TP53* wild type and the *TP53* mutated cell lines and patient samples. This suggests that the Y5 antibody binds to wild type p53, and illustrates the importance of careful validation of the antibodies. However, a recent report demonstrated the presence of p53 protein with “pseudo-mutant” conformation in pre-leukemic hematopoietic stem/progenitor cells and in leukemic blast cells in *TP53* wild type patients [52]. This was shown using a combination of p53 conformation-specific antibodies by mass cytometry. The epitope of the Y5 antibody is in p53 aa 1–100. By personal communication with the vendor (Abcam), we were informed that the antibody detects all mutant forms of p53, including mutations outside the 1–100 aa region, thus presumably recognizing the mutant conformation of the protein. We cannot exclude that the presence of p53 protein with pseudo-mutant conformation is causing the Y5 signal in the p53 wild type cells investigated here, and further experiments are necessary to evaluate this.

The work presented here is focused on the application of mass cytometry in assessment of hematological malignancies. Although we argue that peripheral blood and bone marrow are especially suitable samples for this application, the method is by no means limited to samples of such origin. Several publications presenting mass cytometry analyses of single cell samples originating from solid tissues have been published [53,54,55]. We do, however, emphasize that the required process of disrupting the extracellular matrix and structural proteins to bring the sample into suspension might affect the phosphorylated epitopes. Of note, an imaging-based mass cytometry tool is also available. This novel tool, the Hyperion Imaging System, combines properties of mass cytometry with immunohistochemistry and immunocytochemistry to allow analyses of tissue sections without the need for tissue degradation, and with the additional advantage of preserving tissue architecture [56]. This could be a better alternative for mass cytometry-based investigation of the p53 signaling pathway in solid tissues. However, as a primary solid tumor can metastasize to distant organs through the circulation, hematogenous spread of cancer cells will lead to what is known as circulating tumor cells. In these instances, the metastasizing cells can be found by standard blood sampling, and in cases of adequate amount of these circulating cells combined with a properly designed antibody panel for the tumor in question, mass cytometry has the potential to detect these cells [57].

In summary, mass cytometry has a great potential for p53-centric investigation of primary patient samples. It allows for thorough investigation of surface markers, enabling broad immunophenotyping combined with intracellular signaling proteins, far exceeding current routine methods such as flow cytometry. As precision medicine is emerging and the span of patients eligible for this treatment approach is expanding, increased knowledge of the disease state in every individual patient is of importance and can potentially provide clinical information relevant for future treatment choices. In this work, we demonstrated how currently available mass cytometry technology can be applied to investigate the p53 network in primary clinical samples. Development of p53 isoform-specific antibodies as well as modification- and conformation-specific antibodies compatible with the mass cytometry platform would add increasing value to experiments focusing on p53 and its functions in different malignancies.

## 4. Materials and Methods

This study has been performed in accordance with the Declaration of Helsinki and in agreement with approved local protocols. The study was approved by the regional ethics committee; Regionale komiteer for medisinsk og helsefaglig forskningsetikk (REK) Vest, University of Bergen, Bergen, Norway (Project REK2012/2245). Before study initiation, all subjects provided written informed consent.

Blood samples were collected by phlebotomy of a peripheral vein or drawn from a Hickman central venous catheter into EDTA vacutainers (BD, Franklin Lakes, NJ, USA). Red blood cells were lysed, and white blood cells were fixed using BD lyse/fix Buffer 5X (7.15% methanol (*w*/*w*), 20.35% formaldehyde (*w*/*w*) and 15.65% diethylene glycol (*w*/*w*)) according to manufacturer’s protocol. The samples were stored at −80 °C in NaCl 0.9%. Bone marrow from the HCL patients was aspirated by standard medical procedure into syringes containing heparin, and further sample processing was performed as described for peripheral blood.

MOLM13, NB4 and HL-60 cells were purchased from Deutsche Sammlung von Mikrooranismen und Zellkulturen GmbH (DSMZ, Braunschweig, Germany). OV-90 and Raji cells were purchased from American Type Culture Collection (ATCC, Manassas, VA, USA). All cell lines were cultured in RMPI-1640 medium (Sigma-Aldrich, Inc. St Louis, MO, USA) supplemented with 10% heat inactivated Fetal Bovin Serum (PAA Laboratories GmbH, Pasching, Austria), 1% L-arginine (Sigma-Aldrich) and 1% Penicillin-streptomycin (Sigma-Aldrich), in incubators at 37 °C and 5% CO_2_. For the suspension cells, culture medium was removed by wash with 4 °C NaCl and centrifugation, followed by addition of 4% PFA in PBS and incubation at 37 °C for 10 min. The adherent cell line OV-90 was trypsinized to bring the cells into suspension prior to fixation. Fixed cell lines were stored at −80 °C in NaCl until further processing. MOLM13 cells were irradiated by 25 Gy followed by incubation at 37 °C, 5% CO_2_ for 4 hours, and thereafter fixated by 4% PFA.

The details of the mass cytometry antibody panel are described in Table S2. The p53 antibodies p53 (DO-7), ac-p53 [K382] (REA529) and p53^mut^ (Y5) were purchased from Cell Signaling Technology (Danvers, MA, USA), Miltenyi Biotec (Bergisch Gladbach, Germany) and Abcam (Cambridge, UK), respectively. These antibodies were ordered as custom formulation suspended in BSA- and azide free buffer. This is important for the mass cytometry technology, as carrier BSA will compete with the antibody for binding to the metal tags during conjugation. The antibodies were conjugated to the metal tags according to Fluidigm’s MaxPar Antibody Labeling Kit protocol (MaxPar Antibody Labeling Kit, version 7, South San Francisco, CA, USA). In the conjugation process, the antibodies are covalently bound to a polymer. The polymer includes a chelating group which forms a non-covalent non-reversible interaction with the metal.

The p53-specific antibodies were titrated and validated using appropriate cell line and primary cell controls, as described in the results section. Antibody titrations were performed by five 1:1 serial dilution of the antibodies, starting at 1:50 dilution. Five equal barcoded cell aliquots were stained with the resultant titration series. During titration, all channels susceptible for potential spillover were kept empty, in order to evaluate the actual signal spillover the concentrations tested.

All samples included in the validation and titration were barcoded as described in Fluidigm’s MaxPar Cell ID protocol (PRD 023 V1). After barcoding, the cells were stained with extracellular and intracellular antibodies according to the Fluidigm Phospho Protein staining protocol (PN 400278 A4), with some minor adjustments. This includes the addition of two 20 min incubation steps with 200IU heparin (Wockhardt, Wrexham, UK) immediately preceding the addition of the extracellular and intracellular antibody cocktails. The final heparin concentration during antibody incubation was 100IU. This was done to block unspecific antibody binding by eosinophils, as previously described [58]. In addition, Fc receptor blocking was performed prior to extracellular antibody incubation, by addition of human IgG (Octagam, Octapharma, Lachen, Switzerland, 50 mg/mL) diluted 1:1000 in Cell Staining Buffer (Fluidigm, South San Francisco, CA, USA) incubated for 10 min at room temperature. All antibody incubations were performed with constant agitation to ensure even suspension of the cell and antibody solution throughout the incubation time. After staining was complete, samples were left for over-night incubation at 4 °C with iridium 191 and 193. The next day, samples were washed with Cell Staining Buffer and MaxPar water (Fluidigm), following the Fluidigm Phospho Protein staining protocol. The sample was suspended in EQ Four Elemental Calibration Beads (Fluidigm) and Cell Acquisition solution (Fluidigm) (1:8), and run in a Helios mass cytometer. Prior to sample run, the mass cytometer was tuned using the commercially available tuning solution from Fluidigm. The acquired data (.FCS files) was normalized to the EQ bead standard to correct for potential signal variation over time, and concatenated using the Premessa software. The CATALYST R package was used to debarcode the concatenated sample. Data analysis was performed using CytoBank (Santa Clara, CA, USA) and VorteX (nolanlab/vortex GitHub) (x-shift). The x-shift algorithm uses weighted *k*-nearest neighbor density estimation to form cell clusters, using a range of different *k*-values. The optimal *k*-value (number of clusters) was found using the “elbow point/switch point”. To increase biological interpretability, similar clusters were manually merged. We visualized the data by a force directed layout, which separates the clusters according to relative similarity. Unsupervised clustering using x-shift and graphical representation of the data was performed using the VorteX stand-alone software, downloaded from nolanlab/vortex GitHub.

## 5. Conclusions

In this work, we demonstrate that the p53 protein, the non-functional protein of a mutated *TP53* gene, and the post-translational modification by acetylation at Lysine 382 can be detected by mass cytometry using antibody probes. We further demonstrate the potential of the technology by analyzing patient samples, including assessment of p53 expression in specific cellular subsets. Mass cytometry allows detection of multiple parameters simultaneously, enabling mapping of the p53 network. Thus, these results demonstrate that mass cytometry is a promising tool, which could reveal novel insight into p53 biology in multiple malignancies. Future development of p53 isoform-specific antibodies compatible with this technology could have the potential to further advance the field of p53 research by mass cytometry.

## Figures and Tables

**Table 1:** Overview of the cell lines used and the *TP53* status of the cell lines.

Cell Line	Cell Type	p53 Status
MOLM13	AML, M5	Wild type
NB4	AML, M3	Missense mutation, R248Q [25]
OV-90	Epithelial	Missense mutation, S215R [26]
Raji	Lymphoblast, Burkitt Lymphoma	Mutation, R213Q [27] and an Arg to Pro polymorphism at amino acid 72 [28]
HL-60	AML, M2	-/-

## Supplementary Materials

The following are available online at https://www.mdpi.com/2072-6694/12/12/3699/s1, Figure S1: Visualization of the individual samples by viSNE, Table S1: TP53 status of the primary samples, Table S2: Overview of antibody panel used for patient samples, Table S3: Raw value of surface maker expression (arcsinh transformed dual counts) and the distribution of cell types in the individual samples (percent of total).

## References

[1] Hafner A., Bulyk M.L., Jambhekar A., Lahav G. (2019). The multiple mechanisms that regulate p53 activity and cell fate. Nat. Rev. Mol. Cell Biol..

[2] Riley T., Sontag E.D., Chen P., Levine A.J. (2008). Transcriptional control of human p53-regulated genes. Nat. Rev. Mol. Cell Biol..

[3] Taylor W.R., Stark G.R. (2001). Regulation of the G2/M transition by p53. Oncogene.

[4] Nag S., Qin J., Srivenugopal K.S., Wang M., Zhang R. (2013). The MDM2-p53 pathway revisited. J. Biomed. Res..

[5] Pfister N.T., Prives C. (2017). Transcriptional Regulation by Wild-Type and Cancer-Related Mutant Forms of p53. Cold Spring Harb. Perspect. Med..

[6] Leroy B., Anderson M., Soussi T. (2014). TP53 Mutations in Human Cancer: Database Reassessment and Prospects for the Next Decade. Hum. Mutat..

[7] Short N.J., Rytting M.E., Cortes J.E. (2018). Acute myeloid leukaemia. Lancet.

[8] Ånensen N., Hjelle S.M., Van Belle W., Haaland I., Silden E., Bourdon J.-C., Hovland R., Taskén K., Knappskog S., Lønning P.E. (2012). Correlation analysis of p53 protein isoforms with NPM1/FLT3 mutations and therapy response in acute myeloid leukemia. Oncogene.

[9] Appella E., Anderson C.W. (2001). Post-translational modifications and activation of p53 by genotoxic stresses. Eur. J. Biochem..

[10] Meek D.W. (2015). Regulation of the p53 response and its relationship to cancer1. Biochem. J..

[11] Raffone A., Travaglino A., Cerbone M., De Luca C., Russo D., Di Maio A., De Marco M., Turco M.C., Insabato L., Zullo F. (2020). Diagnostic accuracy of p53 immunohistochemistry as surrogate of TP53 sequencing in endometrial cancer. Pathol. Res. Pract..

[12] Xue Y., Luis B.S., Lane D.P. (2019). Intratumour heterogeneity of p53 expression; causes and consequences. J. Pathol..

[13] Saft L., Karimi M., Ghaderi M., Matolcsy A., Mufti G.J., Kulasekararaj A., Göhring G., Giagounidis A., Selleslag D., Muus P. (2014). p53 protein expression independently predicts outcome in patients with lower-risk myelodysplastic syndromes with del(5q). Haematologica.

[14] Bernard E., Nannya Y., Hasserjian R.P., Devlin S.M., Tuechler H., Medina-Martinez J.S., Yoshizato T., Shiozawa Y., Saiki R., Malcovati L. (2020). Implications of TP53 allelic state for genome stability, clinical presentation and outcomes in myelodysplastic syndromes. Nat. Med..

[15] Fernandez-Pol S., Ma L., Ohgami R.S., Arber D.A. (2016). Immunohistochemistry for p53 is a useful tool to identify cases of acute myeloid leukemia with myelodysplasia-related changes that are TP53 mutated, have complex karyotype, and have poor prognosis. Mod. Pathol..

[16] Tanner S.D., Baranov V.I., Ornatsky O.I., Bandura D.R., George T.C. (2013). An introduction to mass cytometry: Fundamentals and applications. Cancer Immunol. Immunother..

[17] Ornatsky O., Bandura D., Baranov V., Nitz M., Winnik M.A., Tanner S. (2010). Highly multiparametric analysis by mass cytometry. J. Immunol. Methods.

[18] Spitzer M.H., Nolan G.P. (2016). Mass Cytometry: Single Cells, Many Features. Cell.

[19] McCarthy R.L., Mak D.H., Burks J.K., Barton M.C. (2017). Rapid monoisotopic cisplatin based barcoding for multiplexed mass cytometry. Sci. Rep..

[20] Zunder E.R., Lujan E., Goltsev Y., Wernig M., Nolan G.P. (2015). A Continuous Molecular Roadmap to iPSC Reprogramming through Progression Analysis of Single-Cell Mass Cytometry. Cell Stem Cell.

[21] Hartmann F.J., Simonds E.F., Vivanco N., Bruce T., Borges L., Nolan G.P., Spitzer M.H., Bendall S.C. (2019). Scalable Conjugation and Characterization of Immunoglobulins with Stable Mass Isotope Reporters for Single-Cell Mass Cytometry Analysis. Methods Mol. Biol..

[22] Gullaksen S., Bader L., Hellesøy M., Sulen A., Fagerholt O.H.E., Engen C.B., Skavland J., Gjertsen B.T., Gavasso S. (2019). Titrating Complex Mass Cytometry Panels. Cytom. Part A.

[23] Fei P., El-Deiry W.S. (2003). P53 and radiation responses. Oncogene.

[24] Haaland I., Opsahl J.A., Berven F.S., Reikvam H., Fredly H., Haugse R., Thiede B., McCormack E., Lain S., Bruserud Ø. (2014). Molecular mechanisms of nutlin-3 involve acetylation of p53, histones and heat shock proteins in acute myeloid leukemia. Mol. Cancer.

[25] Allende-Vega N., Krzywinska E., Orecchioni S., López-Royuela N., Reggiani F., Talarico G., Rossi J.-F., Rossignol R., Hicheri Y., Cartron G. (2015). The presence of wild type p53 in hematological cancers improves the efficacy of combinational therapy targeting metabolism. Oncotarget.

[26] Bourgeois D.L., Kabarowski K.A., Porubsky V.L., Kreeger P.K. (2015). High-grade serous ovarian cancer cell lines exhibit heterogeneous responses to growth factor stimulation. Cancer Cell Int..

[27] Pan Y., Haines D.S. (2000). Identification of a tumor-derived p53 mutant with novel transactivating selectivity. Oncogene.

[28] Farrell P.J., Allan G.J., Shanahan F., Vousden K.H., Crook T. (1991). p53 is frequently mutated in Burkitt’s lymphoma cell lines. Embo. J..

[29] Cavalcanti G.B., Scheiner M.A.M., Magluta E.P.S., Vasconcelos F.C., Klumb E.M., Maia R.C. (2010). p53 flow cytometry evaluation in leukemias: Correlation to factors affecting clinical outcome. Cytom. Part B Clin. Cytom..

[30] Zhang J., Shen L., Sun L.-Q. (2015). The regulation of radiosensitivity by p53 and its acetylation. Cancer Lett..

[31] Takahashi C., Au-Yeung A., Fuh F., Ramirez-Montagut T., Bolen C., Mathews W., O’Gorman W.E. (2016). Mass cytometry panel optimization through the designed distribution of signal interference. Cytom. Part A.

[32] Samusik N., Good Z., Spitzer Z.G.M.H., Davis K.L., Nolan G.P. (2016). Automated mapping of phenotype space with single-cell data. Nat. Methods.

[33] Amir E.-A.D., Davis K.L., Tadmor M.D., Simonds E.F., Levine J.H., Bendall S.C., Shenfeld D.K., Krishnaswamy S., Nolan G.P., Pe’Er D. (2013). viSNE enables visualization of high dimensional single-cell data and reveals phenotypic heterogeneity of leukemia. Nat. Biotechnol..

[34] Duckworth A.D., Gherardini P.F., Sykorova M., Yasin F., Nolan G.P., Slupsky J.R., Kalakonda N. (2019). Multiplexed profiling of RNA and protein expression signatures in individual cells using flow or mass cytometry. Nat. Protoc..

[35] Mavropoulos A., Allo B., He M., Park E., Majonis D., Ornatsky O. (2017). Simultaneous Detection of Protein and mRNA in Jurkat and KG-1a Cells by Mass Cytometry. Cytom. Part A.

[36] Bourdon J.-C., Surget S., Khoury M.P. (2013). Uncovering the role of p53 splice variants in human malignancy: A clinical perspective. OncoTargets Ther..

[37] Okorokov A.L., Ponchel F., Milner J. (1997). Induced N- and C-terminal cleavage of p53: A core fragment of p53, generated by interaction with damaged DNA, promotes cleavage of the N-terminus of full-length p53, whereas ssDNA induces C-terminal cleavage of p53. EMBO J..

[38] Bonsing B.A., Corver W.E., Gorsira M.C., van Vliet M., Oud P.S., Cornelisse C.J., Fleuren G.J. (1997). Specificity of seven monoclonal antibodies against p53 evaluated with Western blotting, immunohistochemistry, confocal laser scanning microscopy, and flow cytometry. Cytometry.

[39] Leroy B., Girard L., Hollestelle A., Minna J.D., Gazdar A.F., Soussi T. (2014). Analysis of TP53 Mutation Status in Human Cancer Cell Lines: A Reassessment. Hum. Mutat..

[40] Allende-Vega N., Villalba M. (2019). Metabolic stress controls mutant p53 R248Q stability in acute myeloid leukemia cells. Sci. Rep..

[41] Leipold M.D., Obermoser G., Fenwick C., Kleinstuber K., Rashidi N., McNevin J.P., Nau A.N., Wagar L.E., Rozot V., Davis M.M. (2018). Comparison of CyTOF assays across sites: Results of a six-center pilot study. J. Immunol. Methods.

[42] Van Gassen S., Gaudilliere B., Angst M.S., Saeys Y., Aghaeepour N. (2020). CytoNorm: A Normalization Algorithm for Cytometry Data. Cytom. Part A.

[43] Bringeland G.H., Bader L., Blaser N., Budzinski L., Schulz A.R., Mei H.E., Myhr K.-M., Vedeler C.A., Gavasso S. (2019). Optimization of Receptor Occupancy Assays in Mass Cytometry: Standardization Across Channels with QSC Beads. Cytom. Part A.

[44] Bendall S.C., Nolan G.P., Roederer M., Chattopadhyay P.K. (2012). A deep profiler’s guide to cytometry. Trends Immunol..

[45] Maecker H.T., Harari A. (2015). Immune monitoring technology primer: Flow and mass cytometry. J. Immunother. Cancer.

[46] Tricot S., Meyrand M., Sammicheli C., Elhmouzi-Younes J., Corneau A., Bertholet S., Malissen M., Le Grand R., Nuti S., Luche H. (2015). Evaluating the efficiency of isotope transmission for improved panel design and a comparison of the detection sensitivities of mass cytometer instruments. Cytom. Part A.

[47] Irish J.M., Anensen N., Hovland R., Skavland J., Børresen-Dale A.-L., Bruserud Ø., Nolan G.P., Gjertsen B.T., Garand R., Lode L. (2006). Flt3 Y591 duplication and Bcl-2 overexpression are detected in acute myeloid leukemia cells with high levels of phosphorylated wild-type p53. Blood.

[48] Rossi D., Khiabanian H., Spina V., Ciardullo C., Bruscaggin A., Famà R., Rasi S., Monti S., Deambrogi C., De Paoli L. (2014). Clinical impact of small TP53 mutated subclones in chronic lymphocytic leukemia. Blood.

[49] Prochazka K.T., Pregartner G., Rücker F.G., Heitzer E., Pabst G., Wölfler A., Zebisch A., Berghold A., Döhner K., Sill H. (2019). Clinical implications of subclonal TP53 mutations in acute myeloid leukemia. Haematologica.

[50] Abbas T., Dutta A. (2009). p21 in cancer: Intricate networks and multiple activities. Nat. Rev. Cancer.

[51] Prokocimer M., Molchadsky A., Rotter V. (2017). Dysfunctional diversity of p53 proteins in adult acute myeloid leukemia: Projections on diagnostic workup and therapy. Blood.

[52] Tuval A., Kaushansky N., Azogy H., Leshkowitz D., Salame T.M., Minden M.D., Tal P., Rotter V., Oren M., Shlush L. Functional characterization of pre-leukemic hematopioetic cells. Proceedings of the 25th EHA Congress—The European Hematology Association, Virtual Platform.

[53] Poláková I., Pelák O., Thürner D., Pokrývková B., Tachezy R., Kalina T., Smahel M. (2019). Implementation of Mass Cytometry for Immunoprofiling of Patients with Solid Tumors. J. Immunol. Res..

[54] Chevrier S., Levine J.H., Zanotelli V.R.T., Silina K., Schulz D., Bacac M., Ries C.H., Ailles L., Jewett M.A.S., Moch H. (2017). An Immune Atlas of Clear Cell Renal Cell Carcinoma. Cell.

[55] Leelatian N., Doxie D.B., Greenplate A.R., Mobley B.C., Lehman J.M., Sinnaeve J., Kauffmann R.M., Werkhaven J.A., Mistry A.M., Weaver K.D. (2016). Single cell analysis of human tissues and solid tumors with mass cytometry. Cytom. Part B Clin. Cytom..

[56] Giesen C., Wang H.A.O., Schapiro D., Zivanovic N., Jacobs A., Hattendorf B., Schüffler P.J., Grolimund D., Buhmann J.M., Brandt S. (2014). Highly multiplexed imaging of tumor tissues with subcellular resolution by mass cytometry. Nat. Methods.

[57] Micalizzi D.S., Maheswaran S., Haber D.A. (2017). A conduit to metastasis: Circulating tumor cell biology. Genes Dev..

[58] Rahman A.H., Tordesillas L., Berin M.C. (2016). Heparin reduces nonspecific eosinophil staining artifacts in mass cytometry experiments. Cytom. Part A.

